# The miR-641-STIM1 and SATB1 axes play important roles in the regulation of the Th17/Treg balance in ITP

**DOI:** 10.1038/s41598-024-61660-9

**Published:** 2024-05-16

**Authors:** Hongkai Zhu, Xueqin Ruan, Kexin Zhao, Wenyong Kuang, Sufang Liu, Wenzhe Yan, Xianming Fu, Zhao Cheng, Ruijuan Li, Hongling Peng

**Affiliations:** 1 Department of Hematology, The Second Xiangya Hospital, Central South University, No. 139# Renmin Road, Changsha, 410011 Hunan China; 2 Department of Hematology, The Affiliated Children’s Hospital of Xiangya School of Medicine, Central South University, Changsha, Hunan China; 3 Department of Cardiovascular Surgery, The Second Xiangya Hospital, Central South University, Changsha, Hunan China; 4 Department of Hematology, The People’s Hospital of Guangxi Zhuang Autonomous Region, Guangxi Academy of Medical Sciences, P.R. China, Nanning, China

**Keywords:** Immune thrombocytopenia (ITP), Th17/Treg balance, microRNA 641 (miR-641), SATB1, STIM1, Cell biology, Immunology, Molecular biology

## Abstract

Immune thrombocytopenia (ITP) is an autoimmune disease caused by T-cell dysfunction. Recently, several studies have shown that a disturbed Th17/Treg balance contributes to the development of ITP. MicroRNAs (miRNAs) are small noncoding RNA moleculesthat posttranscriptionally regulate gene expression. Emerging evidences have demonstrated that miRNAs play an important role in regulating the Th17/Treg balance. In the present study, we found that miR-641 was upregulated in ITP patients. In primary T cells, overexpression of miR-641 could cause downregulation of its target genes STIM1 and SATB1, thus inducing a Th17 (upregulated)/Treg (downregulated) imbalance. Inhibition of miR-641 by a miR-641 sponge in primary T cells of ITP patients or by antagomiR-641 in an ITP murine model could cause upregulation of STIM1 and SATB1, thus restoring Th17/Treg homeostasis. These results suggested that the miR-641-STIM/SATB1 axis plays an important role in regulating the Th17/Treg balance in ITP.

## Introduction

Immune thrombocytopenia (ITP) is an acquired autoimmune disease. It is the most common hemorrhagic disorder caused by a decrease in platelet count. The pathogenesis of ITP is complex and involves multiple factors. The destruction of platelets in ITP occurs primarily through two mechanisms: antibody-mediated clearance by the spleen and immune-mediated damage to megakaryocytes. The currently known T cells dysfunctions of ITP includes T-cell toxicity^1^, abnormal regulatory T cells^2–4^, an imbalance in helper T cells^5,6^, abnormal T-cell responses^7,8^. A growing body of research suggests that the Treg/Th17 balance plays a key role in the development of ITP^4,9–11^. Resveratrol^9^, indirubin^12,13^, and dexamethasone^14,15^ reportedly modulate Th17/Treg homeostasis.

In addition to traditional drug therapy, research into the role of noncoding RNAs in the pathogenesis of ITP has been intensively conducted in recent years^16–18^. microRNAs (miR-106p-5p, miR99a, miR-182-5p and miR-183-5P), long noncoding RNA MEG3, and lncRNA PVT1have been shown to play important roles in regulating Th17/Treg imbalance in CD4+ cells^11,19–21^ in autoimmune diseases. These studies emphasized the important roles of noncoding RNA functions in regulating T-cell functions.

Since T-cell functions are important in the development of ITP^22^ and since noncoding RNAs, especially miRNAs, play important roles in immune regulation^18^, we propose that miRNAs have the potential to regulate the development of ITP. We observed that microRNA 641 (miR-641) and its potential target genes SATB1 and STIM1 were differentially expressed in the peripheral T cells of ITP patients compared with those of normal candidates. They may function as potential targets for regulating the Th17/Treg balance. Extensive investigations were conducted to elucidate the role of the miR-641-SATB1 and STIM1 axis in ITP both in vitro and in vivo.

## Methods

### Patients and samples

Patients were diagnosed with ITP according to the American Society of Hematology guidelines^23–26^. Peripheral blood samples from ITP patients (n = 7) and normal candidates (n = 5) were collected after informed written consent was obtained in accordance with the Declaration of Helsinki (Table 1). This study was approved by the institutional ethics committee of The Second Xiangya Hospital, Central South University.

**Table 1:** ITP Patients and Normal Candidates.

ITP	SEX	AGE	WBC (10^9^/L)	RBC (10^12^/L)	Platelet (10^9^/L)	Hb (g/L)	Course of disease (years)
q-PCR (miR641, SATB1, STIM1, TGFBR1, DDX5) Peripheral T cells	Female	48	7.41	4.02	15	123	20+
q-PCR (miR641, SATB1, STIM1, TGFBR1, DDX5) Peripheral T cells	Male	55	8.98	4.8	9	149	Newly diagnosis
q-PCR (miR641, SATB1, STIM1, TGFBR1, DDX5)	Male	65	11.42	5.2	14	159	10+
q-PCR (miR641, SATB1, STIM1) Peripheral T cells	Female	36	5.22	4.51	38	141	0.5
q-PCR (miR641, SATB1, STIM1)	Female	62	9.27	4.65	20	138	0.3
q-PCR (miR641 only)	Female	64	7.52	4.3	27	124	0.5
q-PCR	Male	37	4.5	5.25	4	124	0.7
(miR641 only)							
Normal Candidate	SEX	AGE	WBC(10^9^/L)	RBC (10^12^/L)	Platelet (10^9^/L)	Hb (g/L)	Course of disease (years)
q-PCR (miR641, SATB1, STIM1,TGFBR1, DDX5) Peripheral T cells	Male	22	5.77	4.04	226	155	/
q-PCR (miR641, SATB1, STIM1,TGFBR1, DDX5) Peripheral T cells	Female	58	7.69	4.56	189	128	/
q-PCR (miR641, SATB1, STIM1,TGFBR1, DDX5) Peripheral T cells	Male	27	5.93	4.38	277	146	/
q-PCR (miR641, SATB1, STIM1)	Male	25	6.71	4.88	209	139	/
q-PCR (miR641, SATB1, STIM1)	Female	46	5.32	4.99	208	124	/

### Pan-T-cell isolation

Pan-T cells were isolated using a Pan-T-cell isolation kit, human (Miltenyi Biotec, 130–096-535) or a Pan-T-cell isolation kit, mouse (Miltenyi Biotec, 130-095-130) following the manufacturer’s instructions. In brief, PBMNCs from peripheral blood were collected using Ficoll Paque Plus (M19059, GE Healthcare). The cells were counted, and the amount of reagents used for subsequent experiments was calculated based on the cell count. The cells were centrifuged at 1200 rpm for 5 min and resuspended in 40 µl of buffer (AutoMACS Running buffer:AutoMACS Risining solution = 1:20) per 10^7 cells. The mixture was transferred to 1.5 ml EP tubes. Then, 10 µl of Pan-T-cell Biotin-Antibody Cocktail was added, mixed well and incubated at 2–8 °C for 5 min. Thirty microliters of buffer was added per 10^7 cells. Ten microliters of Pan-T-cell MicroBead Cocktail was added to each unit, and the mixture was mixed well and incubated at 2–8 °C for 10 min. The LS column and magnetic field were prepared, the column was rinsed with 3 ml of buffer, and the cell suspension was applied to the column. The flow-throughs containing unlabeled cells, representing the enriched T cells, were collected.

### qRT‒PCR (miRNA and mRNA)

For miRNA qRT‒PCR, the miDETECTATrackTM miRNA qRT‒PCR Starter Kit (Guangzhou RiboBio Co., Ltd., China, Inc.) was used to reverse transcribe the total RNA to cDNA andamplify the RT product according to the manufacturer’sinstructions. Theprimer sequences were synthesized by Guangzhou RiboBio Co., Ltd., China, andare summarized in the Supplementary Table. For target gene coding mRNA primer design, the PICK PRIMERS tool in the nucleic acid module of NCBI (https://www.ncbi.nlm.nih.gov/nuccore) was used. qRT‒PCR was performed using the SYBR Green method. Reverse transcription into cDNA was performed using random primers and quantified by SYBR Green (Table S1). All groups underwent three technical replicates.

### Luciferase assay

HEK-293 T (293 T) were obtained from American Type Culture Collection (ATCC). Cells were cultured to the logarithmic growth phase, removed and inoculated in 96-well plates (LUMITRAC™ 200 96-well culture plates) at a density of 10,000 cells per well, with 100 µl of medium added to each well, and incubated in a cell culture incubator at 37 °C for one day. OPTI-MEM was supplemented with miRNA mimics (micrONTM hsa-miR-641 mimic and micrONTM miRNA mimic negative control #24; Guangzhou RiboBio Co., Ltd., China), target controls, 3' untranslated region double reporter vectors of the target gene or mutation vectors (the specific sequences are described in Supplementary Material Table S4 & S5) diluted in Lipo 6000™ transfection reagent (Beyotime Biotechnology, China) in Opti-MEM at diluted concentrations, after which the constructs were allowed to stand and mix. The concentration of the transfected mimics was set at 50 nM, and the concentration of the plasmid was maintained at 50 ng per well. Three replicate wells were set up and changed after 6 h. The luciferase substrate and luciferase buffer from the kit (Dual-Glo® Luciferase Assay System, Promega) were mixed. The stop & Glo buffer was brought to room temperature before use, and the appropriate amount of stop & Glo substrate was added to the stop & Glo buffer. After 2 days of transfection, the medium was aspirated, 35 μL of 1X PBS was added to each well, 35 μL of luciferase reagent was added to each well, and the mixture was shaken and aspirated into a 96-well plate for fluorescence analysis. Then, 30 μL of Stop reagent was added to each well, the plate was shaken for 10 min, and the fluorescence was measured using aspectrophotometer. All groups underwent three technical replicates.

### Plasmid construction for miR-641 OE or miR-641 sponge

The miR-641 overexpression and miR-641sponge plasmids were constructed (HANBIO Biotechnology (Shanghai) Co.) (see Table S2, S3 for plasmid structure and sequence details), and polybrene was used for the viral transfection enhancement solution. For primary T-cell transfection, the MOI was set to 100, and for cell line transfection, the MOI was set to 50. Primary T-cell transfection was performed in 6-well plates, while cell lines were used in 24-well plates.

### Activation of primary T cells

Non-tissue-treated 6-well plates were prepacked with 5 μg/ml anti-CD3 antibody and 1 μg/ml soluble anti-CD28 antibody. Peripheral T cells were then grown in 6-well plates in RPMI 1640 medium supplemented with 10% fetal bovine serum, 100 U/ml penicillin and 50 μg/ml streptomycin. Cell differentiation was induced by the appropriate cytokines and antibodies. For the induction of Th17 differentiation, IL-6 (10 ng/ml), IL-1β (10 ng/ml), TGF-β1 (10 ng/ml), IL-23 (10 ng/ml), anti-IL-4 (10 μg/ml) and anti-IFN-γ (10 μg/ml) were added to the medium. For the induction of Treg differentiation, the medium was supplemented with 300 U/ml IL-2, 10 ng/ml TGF-β1, 10 μg/mL anti-IL-4 and 10 μg/mL anti-IFN-γ. The cells were incubated at 37 °C for 96 h and then collected for further investigations.

### Western blotting

The cells were harvested and homogenized on ice in RIPA buffer (Beyotime Biotechnology P0013B) containing proteinase inhibitor (complete EDTA-free protease inhibitor cocktail tablets provided in EASY packs, Roche, 04 693 132 001). Equivalent amounts of cell lysate were subjected to sodium dodecyl sulfate‒polyacrylamide gel electrophoresis (SDS‒PAGE) and then transferred to polyvinylidene fluoride (PVDF) membranes for Western blotting. The membranes were incubated with an anti-SATB1 antibody ([EPR3951] (ab109122, Abcam), an anti-Stromal interaction molecule 1 antibody (STIM1) ([EPR3414] (ab108994), an anti-β-Actin monoclonal antibody (66009-1-Ig, Proteintech), and appropriate horseradish peroxidase (HRP)-linked anti-rabbit/mouse IgG secondary antibodies (SA00001-1, SA00001-2, Proteintech) according to the primary antibody species. The membranes were treated with an enhanced chemiluminescent detection kit (Thermo Fisher Scientific, #17097), and the signals were collected and photographed (Bio-Rad, Gel Doc, XR). All experiments were repeated 3 times. The bands were quantified by densitometry with Scion Image software (ImageJ 1.42q; NIH, Bethesda, MD).

### FACS analysis

The cells were resuspended in 1640 medium, and the corresponding antibodies were added. Human T cells were labeled with APC-conjugated anti-humanCD4 (357407, Biolegend), PE/CY7-conjugated anti-human IL-17a (512315, Biolegend) for Th17 cells, and APC-conjugated anti-humanCD4 (357407, Biolegend), APC/CY7-conjugated anti-human CD25 (302613, Biolegend) and PE-conjugated anti-humanFOXP3 (320207, Biolegend) for Treg cells.Murine T cells were analyzed as follows: Th17 cells were first gated with APC/Fire 750-conjugated anti-mouse CD45 (147713, Biolgend), PerCP/Cyanine5.5-conjugated anti-mouse CD4 (100433, Biolegend), Alexa Fluor 488-conjugated anti-mouse IL-17A(506909, Biolegend), and Tregs were first gated with APC/Fire 750-conjugated anti-mouse CD45 (147713, Biolgend). Then, the cells labeled with anti-mouse CD4 (100433, Biolegend) were selected to identify PE-conjugated anti-mouse FOXP3(126403, Biolegend) and APC-conjugated anti-mouse CD25 (101909, Biolegend) double-positive cells.

IL-17a, which labels Th17 cells, is abundant intracellularly and requires treatment with PBS-Triton solution before labeling.FOXP3 is abundant intracellularly and requires treatment with PBS-Triton solution before labeling.

The percentages of Treg and Th17 cells were then measured using flow cytometry (BD FACS Canto II,BD Biosciences) and analyzed using FlowJo V10 (FlowJo LLC, BD Biosciences).

FACS analysis underwent biological repilication (n = 3).

### Animal guideline

BALB/C mice were purchased from Hunan SJA Laboratory Animal Co.,Ltd., China. The study were performed strictly according to ARRIVE guidelines. Mice were anesthetized or sacrificed according to American Veterinary Medical Association guidelines. All the animal study are approved by the Animal Ethic of The Second Xiangya Hospital, Central South University.

### ITP Murine model

Mice were divided into three groups:the MOCK group (saline 100 µl + BSA1.5 mg/ml) (n = 6), the ITP group (saline 100 µl + BSA 1.5 mg/ml + Mweg30 82.5 µg/ml) (n = 6) and the AntagomiR 641 group (saline 100 µl + BSA 1.5 mg/ml + Mweg30 82.5 µg/ml + AntogomiR 641 5 nmol) (n = 6).

The ITP murine model was induced by intraperitoneal injection of the antiplatelet antibody MWReg30 using a micro-osmotic pump (Alzet micro-osmotic pump, Model 1002; Alza, Palo Alto, CA). The micro-osmotic pump was filled with 100 μl of sterile saline containing MWReg30 (82.5 μg/ml) and bovine serum albumin (1.5 mg/ml) and inserted into the peritoneal cavity of the mice. MWReg30 was released at a rate of 0.5 μl/h for 8 days^27,28^. Mice in the AntagomiR-641 group were injected with the micrOFF hsa-miR-641 antagomir (miR30003311-4, Guangzhou RiboBio Co. Ltd. China) via the tail vein 4 days after the construction of the ITP murine model. The micrOFF™ miRNA antagomir is a specially chemically modified miRNA antagonist that inhibits the action of miRNAs by competitively binding to mature miRNAs in vivo, preventing complementary pairing of miRNAs with their target mRNAs. The miRNA antagomir is the reverse complementary sequence of the mature miRNA strand to the whole strand. They are modified by methylation, with two- and four-base sulfation modifications at the 5' and 3' ends, respectively, and a high-affinity cholesterol modification at the 3' end^29^. All mice were sacrificed on Day 8, and their bone marrow and peripheral blood were collected for further investigations (Fig. 5A).

### Cytokine assay

3 mice in each group were randomly selected for Cytokine Assay. Merck Millipore liquid phase microarray assays were used to detect plasma cytokines. The microspheres are color coded internally using a variety of fluorescent dyes. The precise concentration of these dyes allows the creation of clear colored bead sets of 500 nonmagnetic microspheres (5.6 µm) or 80 magnetic polystyrene microspheres (6.45 µm), each coated with a specific capture antibody. After the beads were captured and analyzed in the test sample, biotinylated detection antibodies were introduced. The reaction mixture was then incubated with the reporter molecule streptavidin PE conjugate to complete the reaction on the surface of each microsphere. Each microsphere was identified, and its bioassay was quantified based on the fluorescent reporter signal. Assays were performed with Luminex® 200TM software. The median fluorescence intensity (MFI) data were saved and analyzed, and the concentration of the protein to be detected in the sample was calculated using a 5-parameter (5pl) fitted curve.

### Giemsa staining

3 mice in each group were randomly selected for Giemsa Staining. Wright‒Giemsa staining was conducted using Wright‒Giemsa staining solution (BA4017A, BASO, China) according to the manufacturer’s instructions. In brief, bone marrow smears were prepared and fixed with absolute methanol for 2 min. The cells were stained with Wright‒Giemsa Stain Solution for 1 min at room temperature. After staining, the slides were rinsed in PBS for 5 min, rinsed in running deionized water (5–6 immersions), and then allowed to air dry. Slides were observed and photographedwith an Axio Scope A1 microscope (Microscopes, Software & Imaging Solutions ZEISS, Germany). The cell types were identified by a pathologist. Giemsa Staining underwent biological repilication (n = 3).

### Platelet count

3 mice in each group were randomly selected for Platelet count. Platelet counts were conducted using a Sysmex XN-1000B1 automated hematology analyzer (Sysmex Europe GmbH, Germany). EDTA-treated peripheral blood was collected from the mice in each group and analyzed following the manufacturer’s instructions. Platelet count analysis underwent biological replication (n = 3). The platelet count was statistically analyzed.

### Statistical analysis

Quantitative data are presented as the mean ± standard error ofthe mean (SEM). Comparisons between groupswere made with one-way ANOVA or the Mann–Whitney U test. Differences were considered significant at *P < 0.05 or ***P < 0.001.

### Statement on guideline and consent

The study was approved by the guidelines institutional ethics committee of The Second Xiangya Hospital, Central South University. All methods were performed in accordance with the guidelines institutional ethics committee of The Second Xiangya Hospital, Central South University. The animal experiment were performed strictly according to ARRIVE guidelines. Mice were anesthetized or sacrificed according to American Veterinary Medical Association guidelines. All the animal study are approved by the Animal Ethic of The Second Xiangya Hospital, Central South University.

### Ethical approval and consent to participate

Patient samples were collected after informed written consent was obtained accordance with the Declaration of Helsinki. This study was approved by the institutional ethics committee of The Second Xiangya Hospital, Central South University.

### Statement

This study was approved by the institutional ethics committee of The Second Xiangya Hospital, Central South University. The study is reported in accordance with ARRIVE guidelines.

## Results

1. Differentially expressed miR-641 and its potential target mRNAs

**Figure 1:**
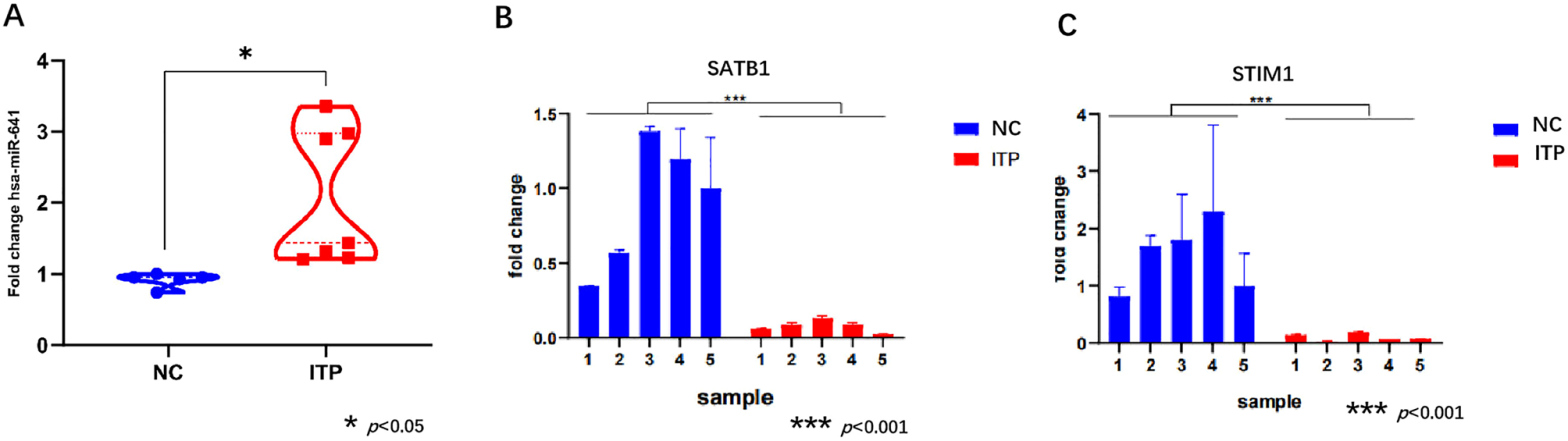
(**A**) qRT-PCR analyzed miR641 expression in normal control (NC, blue, n = 5) and ITP patient (ITP, Red, n = 7), * *p* < 0.05. (**B**) qRT-PCR analyzed SATB1 expression in normal control (NC, blue, n = 5) and ITP patients (ITP, Red, n = 5), *** *p* < 0.001. (**C**) qRT-PCR analyzed STIM1 expression in normal control (NC, blue, n = 5) and ITP patients (ITP, Red, n = 5), *** *p* < 0.001. All groups underwent three technical replicates for qRT-PCR.

qRT‒PCR was performed to investigate the expression of miR-641 and its target genes (SATB1 and STIM1) in the peripheral T cells of ITP patients and in normal candidates (NCs) (Table 1). The results showed that miR-641 expression was upregulated in ITP patients compared with that in NCs (*p* < 0.05) (Fig. 1A).

2. Identifying the potential target genes of miR-641 in the T cells of ITP patients

**Figure 2:**
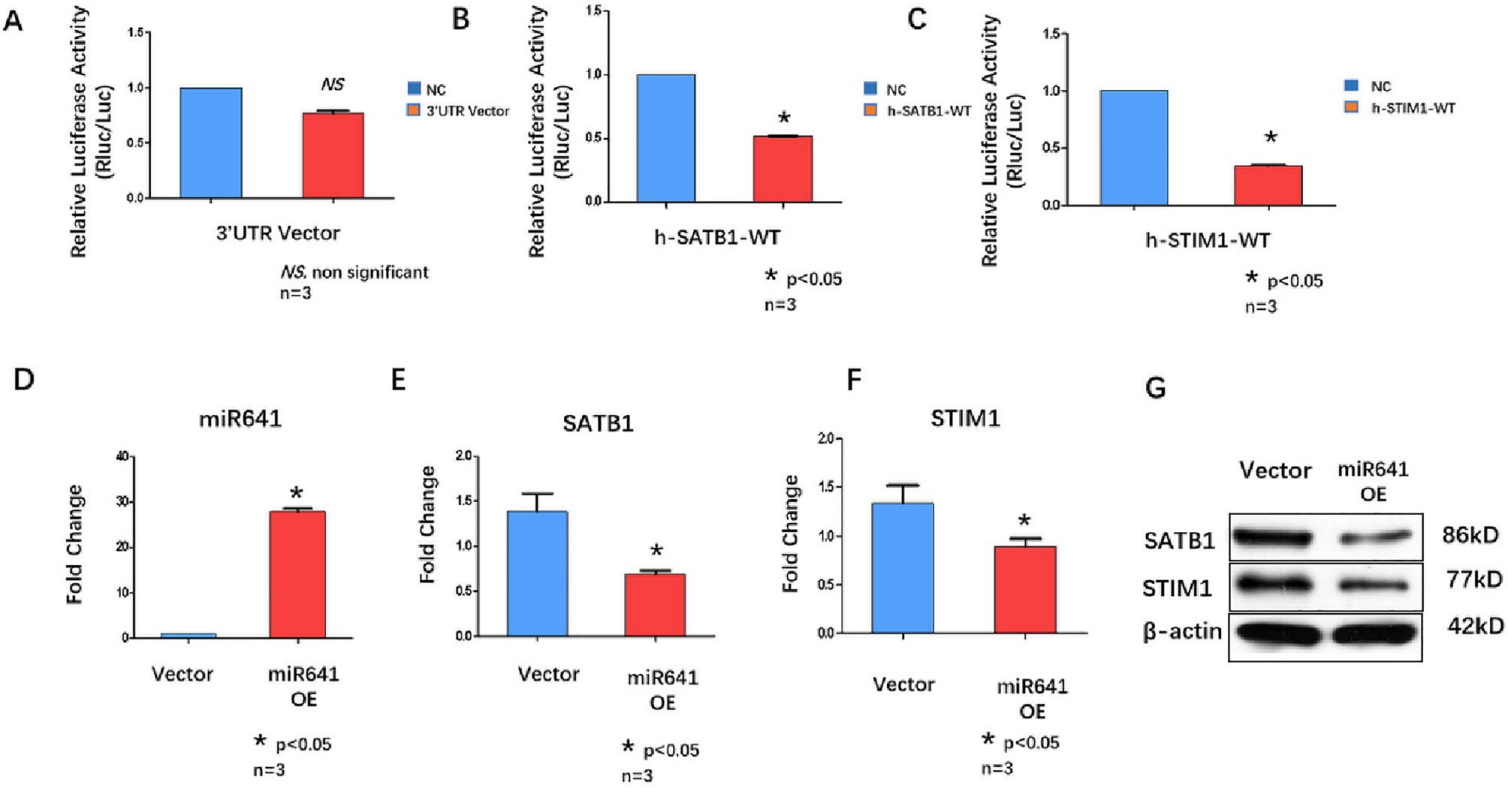
(**A**) Luciferase Assay analyzed the relative Luciferase Activity (NC, blue column; 3’UTR Vector, Red column). *NS.* non significant. (**B**) Luciferase Assay analyzed the relative Luciferase Activity (NC, blue column; h-SATB1-WT, Red column). (**C**) Luciferase Assay analyzed the relative Luciferase Activity (NC, blue column; h-STIM1-WT, Red column). All groups underwent three technical replicates. D. qRT-PCR analyzed miR641 expression in 293 T cells after miR641 over-expression (lentivirus transfection), (Vecter, blue column; miR641 over expression, Red column), *p < 0.05 . E. qRT-PCR analyzed SATB1 expression in 293 T cells after miR641 over-expression(lentivirus transfection), (Vecter, blue column; miR641 over -expression, Red column), *p < 0.05. F. qRT-PCR analyzed STIM1 expression in 293 T cells after miR641 over-expression (lentivirus transfection), (Vecter, blue column; miR641 over- expression, Red column), *p < 0.05 . G. Western blotting analyzed the expression SATB1 and STIM1 encoded proteins after miR641 over-expression (lentivirus transfection) in 293 T cells (Vector Left; miR641 over-expression Right). All groups underwent three technical replicates.

The Mirdb database (http://mirdb.org/database) was used to predict the potential target genes of hsa-miR-641 in both humans and mice. By cross-checking published articles describing important genes correlated with T-cell regulation, 2 potential target genes (SATB1 and STIM1) were selected for further investigation. The results of qRT‒PCR analysis showed that the expression of both SATB1 (*p* < 0.001) and STIM1 (*p* < 0.001) was significantly downregulated in the T cells of ITP patients compared with those of NCs (Fig. 1B,C). Dual-luciferase analysis was further performed to investigate the interaction between miR-641 and its potential target genes (SATB1 and STIM1). We observed a direct interaction of miR-641 with SATB1 and STIM1 (Fig. 2A,B,C).

293 T cells were transfected with lentivirus (miR-641) to overexpress miR-641 (Fig. 2D). The expression of SATB1 and STIM1 was analyzed by qRT‒PCR (Fig. 2E,F), and the expression of their encoded proteins was analyzed by Western blotting (Fig. 2G). The results suggested that overexpression of miR-641 could lead to downregulation of the expression of SATB1 and STIM1 mRNA and their encoded proteins.

3. The overexpression of miR-641 in T cells isolated from normal candidates leads to an imbalance of Th17/Treg cells.

**Figure3:**
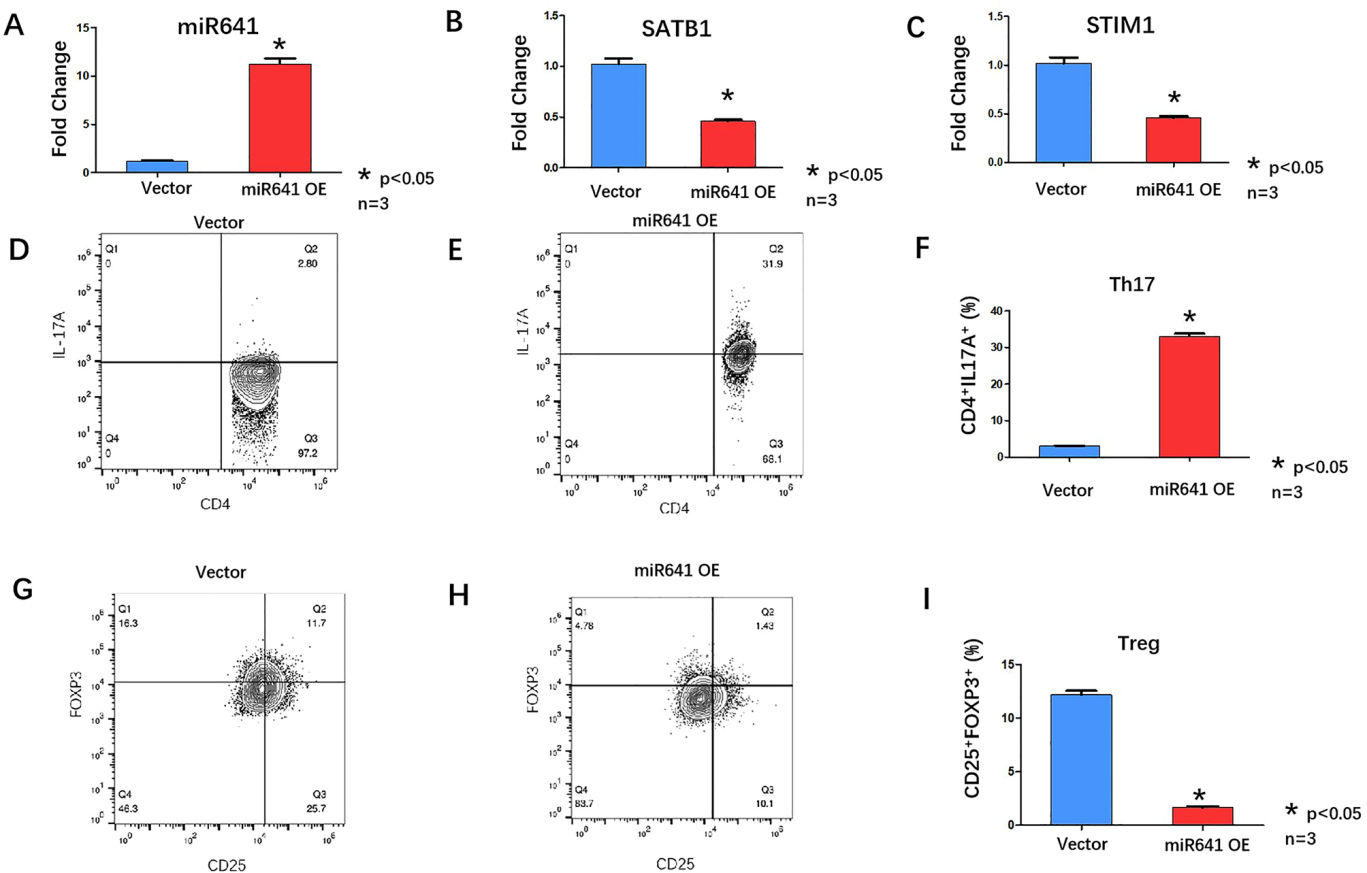
(**A**) qRT-PCR analyzed miR641 expression after miR641 over-expression (lentivirus transfection) in primary T cells from 3 normal candidates. (Vector, blue column; miR641 over-expression, Red Column), * *p* < 0.05. B. qRT-PCR analyzed SATB1 expression after miR641 over-expression (lentivirus transfection) in primary T cells from 3 normal candidates (Vector, blue column; miR641 over-expression, Red Column), *p < 0.05. (**C**) qRT-PCR analyzed STIM1 expression after miR641 over-expression (lentivirus transfection) in primary T cells from 3 normal candidates. (Vector, blue column; miR641 over-expression, Red Column), *p < 0.05. (**D**,**E**) FACS analyzed the Th17 cells surface markers CD4 and IL-17A expression after miR641 over-expression (lentivirus transfection) in primary T cells from 3 normal candidates. (**F**) Statistic analysis of FACS results: CD4^+^IL17A^+^ Th17 cells were up-regulated in miR641 over-expression primary T cells from normal candidates (Red Column) comparing with vector only group (Blue Column). (**G**,**H**) FACS analyzed the Treg cells surface markers CD25 and Foxp3 expression after miR641 over-expression (lentivirus transfection) in primary T cells from 3 normal candidates. (**I**) Statistic analysis of FACS results: CD25^+^Foxp3^+^ cells were up-regulated in miR641 over-expression primary T cells from 3 normal candidates (Red Column) comparing with vector only group (Blue Column).

T cells were isolated from normal candidates using a Pan-T-Cell Isolation Kit (Miltenyi Biotec, 130-096-535)and defined as NC T cells. miR-641 was overexpressed in NC-treated T cells (Fig. 3A). qRT‒PCR was used to analyze the expression of SATB1 and STIM1. The results showed that SATB1 and STIM1 mRNA were downregulated in miR-641-overexpressing NC-treated T cells (Fig. 3B,C). Flowcytometry was applied to investigate the proportions of the Th17 and Treg subpopulations among the CD4^+^ T cells. The number of IL17-expressing cells was increased (2.93 ± 0.13 vs. 33.07 ± 0.76) (Fig. 3D,E,F), while the number of CD4^+^ CD25^+^ Foxp3^+^ Treg cells was decreased in hsa-miR-641-overexpressing NC-T cells (12.17 ± 0.37 vs*.* 1.66 ± 0.13), which led to an imbalance of Th17/Treg cells(Fig. 3G,H,I).

4. Knocking down miR-641 in T cells isolated from ITP patients alleviated the imbalance of Th17/Treg cells

**Figure 4:**
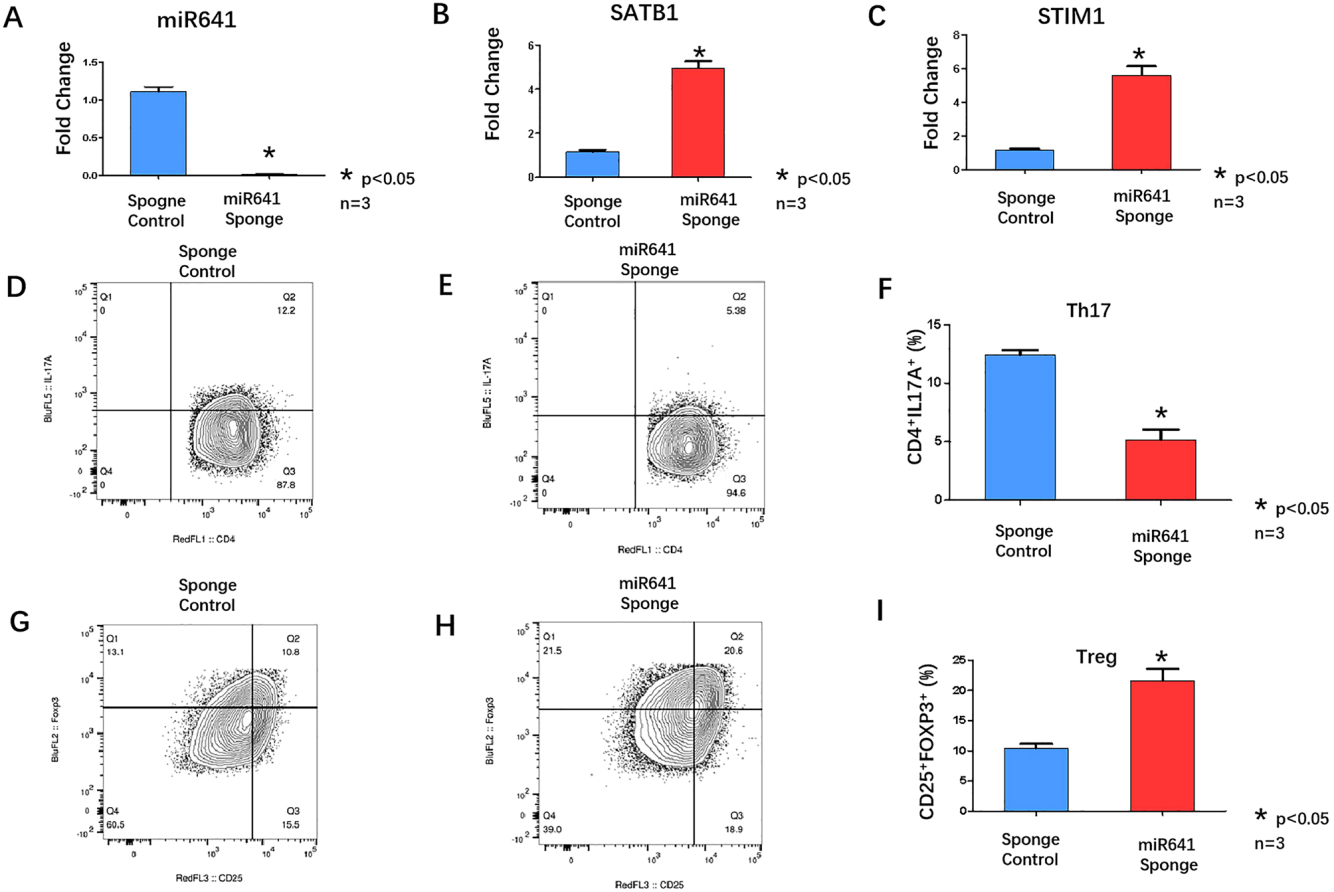
(**A**) qRT-PCR analyzed miR641 expression in miR641sponge transfected primary T cells from 3 ITP patients. (Sponge Control, blue column; miR641 sponge, Red Column), * *p* < 0.05. (**B**) qRT-PCR analyzed SATB1 expression in miR641sponge transfected primary T cells from 3 ITP patients. (Sponge Control, blue column; miR641 sponge, Red Column), * *p* < 0.05. (**C**) qRT-PCR analyzed STIM1 expression in miR641sponge transfected primary T cells from 3 ITP patients. (Sponge Control, blue column; miR641 sponge, Red Column), * *p* < 0.05. (**D**,**E**) FACS analyzed the Th17 cells surface markers CD4 and IL-17A expression in miR641 sponge transfected primary T cells from 3 ITP patients. (**F**) Statistic analysis of FACS results: CD4^+^IL17A^+^ Th17 cells were down-regulated in miR641sponge transfected primary T cells from 3 ITP patients (Red Column) comparing with Sponge Control group (Blue Column). (**G**,**H**) FACS analyzed the Treg cells surface markers CD25 and Foxp3 expression in miR641 sponge transfected primary T cells from 3 ITP patients. (**I**) Statistic analysis of FACS results: CD25^+^Foxp3^+^ Treg cells were up-regulated in miR641sponge transfected primary T cells fromITP patients (Red Column) comparing with Sponge Control group (Blue Column).

T cells were isolated from ITP patients using a Pan T-Cell Isolation Kit human (Miltenyi Biotec, 130–096-535), defined as ITP patient T cells. miR-641 was blocked in ITP patient T cells using a miR-641 sponge (Fig. 4A). q-PCR was used to analyze the expression of SATB1 and STIM1. The results showed that SATB1 and STIM1 mRNA were upregulated in miR-641-blocked ITP patient T cells (Fig. 4B,C). Flowcytometry was applied to investigate the proportions of the Th17 and Treg subpopulations among the CD4^+^ T cells. IL17 expression was downregulated (11.98 ± 0.29 vs. 5.87 ± 0.46) (Fig. 4D,E,F), while CD4^+^ CD25^+^ Foxp3^+^ Treg cells were upregulated in miR-641-blocked ITP patient T cells (10.48 ± 0.71 VS*.* 21.61 ± 1.99), which led to an imbalance in Th17/Treg cells (Fig. 4G,H,I).

5. Intravenous injection of antagomiR-641 restored Th17/Treg balance in a murine ITP model.

**Figure 5:**
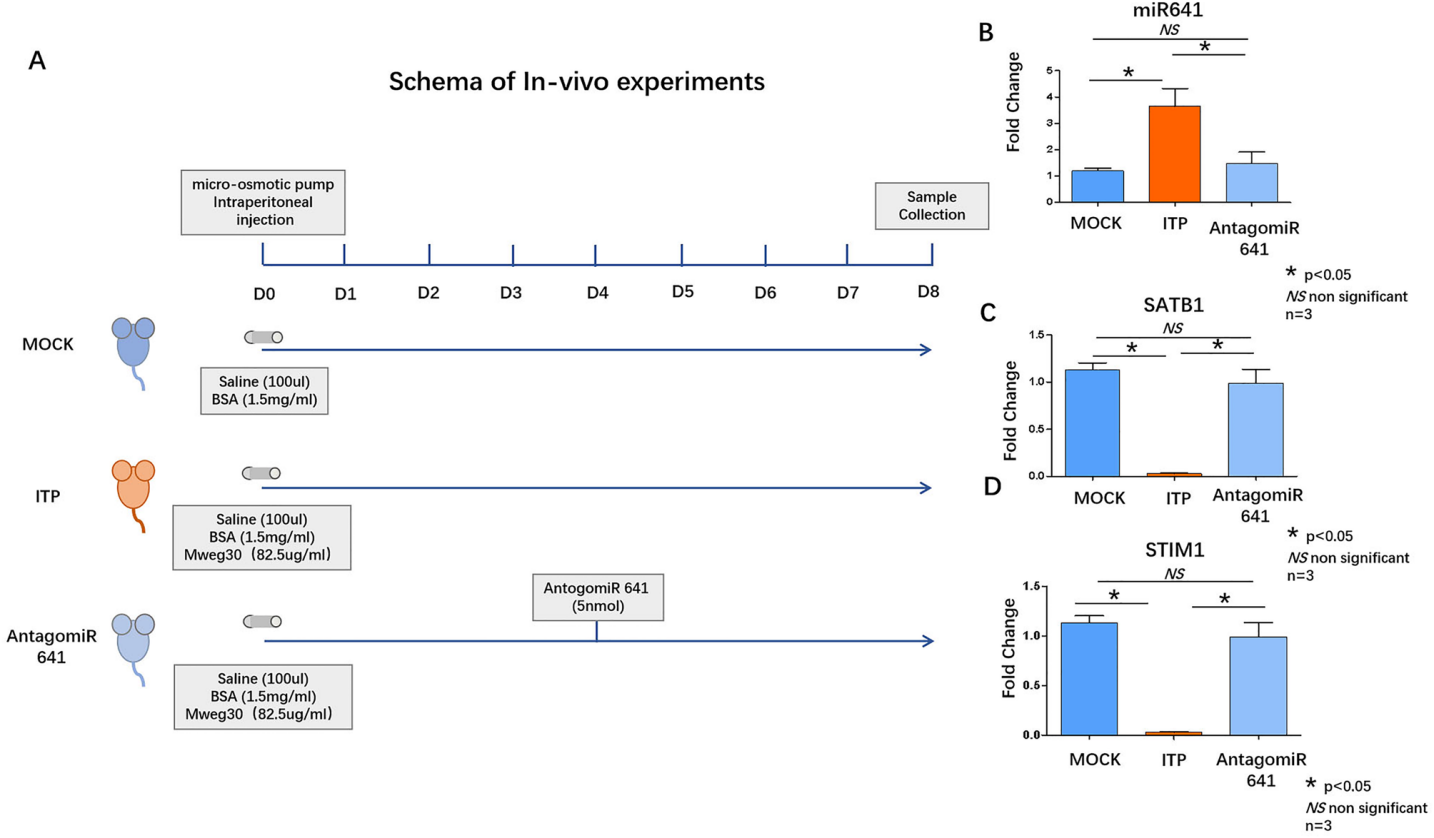
(**A**) Schema of in vivo experiments. (**B**) qRT-PCR analyzed miR641 expression in the peripheral blood from MOCK group (dark blue column), ITP group (Red column) and AntagomiR641group (light blue column). (**C**) qRT-PCR analyzed SATB1 expression in the peripheral blood from MOCK group (dark blue column), ITP group (Red column) and AntagomiR641group (light blue column). (**D**) qRT-PCR analyzed STIM1 expression in the peripheral blood from MOCK group (dark blue column), ITP group (Red column) and AntagomiR641group (light blue column). qRT-PCR analysis underwent biological replication (n = 3).

The murine ITP model was established according to previous reports^27,28^. Mice were divided into the MOCK group (saline (100 µl), BSA (1.5 mg/ml), osmotic pump), ITP group (Mweg30 (82.5 µg/ml), saline (100 µl), BSA(1.5 mg/ml), osmotic pump). The AntagomiR-641 groups (Mweg30 (82.5 µg/ml), saline (100 µl), BSA(1.5 mg/ml), osmotic pump, and AntagomiR-641 (5 nmol, on D4))are shown in the schema (Fig. 5A). Peripheral blood was collected on D8. T cells were isolated using a Pan-T-Cell Isolation Kit II, mouse (Miltenyi Biotec, 130–095-130). qRT‒PCR was used to analyze the expression of miR-641 in each group. The results showed that miR-641 was upregulated in the ITP group, while its expression was blocked in the antagomiR-641 group (Fig. 5B). qRT‒PCR was used to analyze the expression of SATB1 and STIM1 in each group. The results showed that both SATB1 and STIM 1 were downregulated in the ITP group, while they were upregulated in the antagomiR group (Fig. 5C,D).

**Figure 6:**
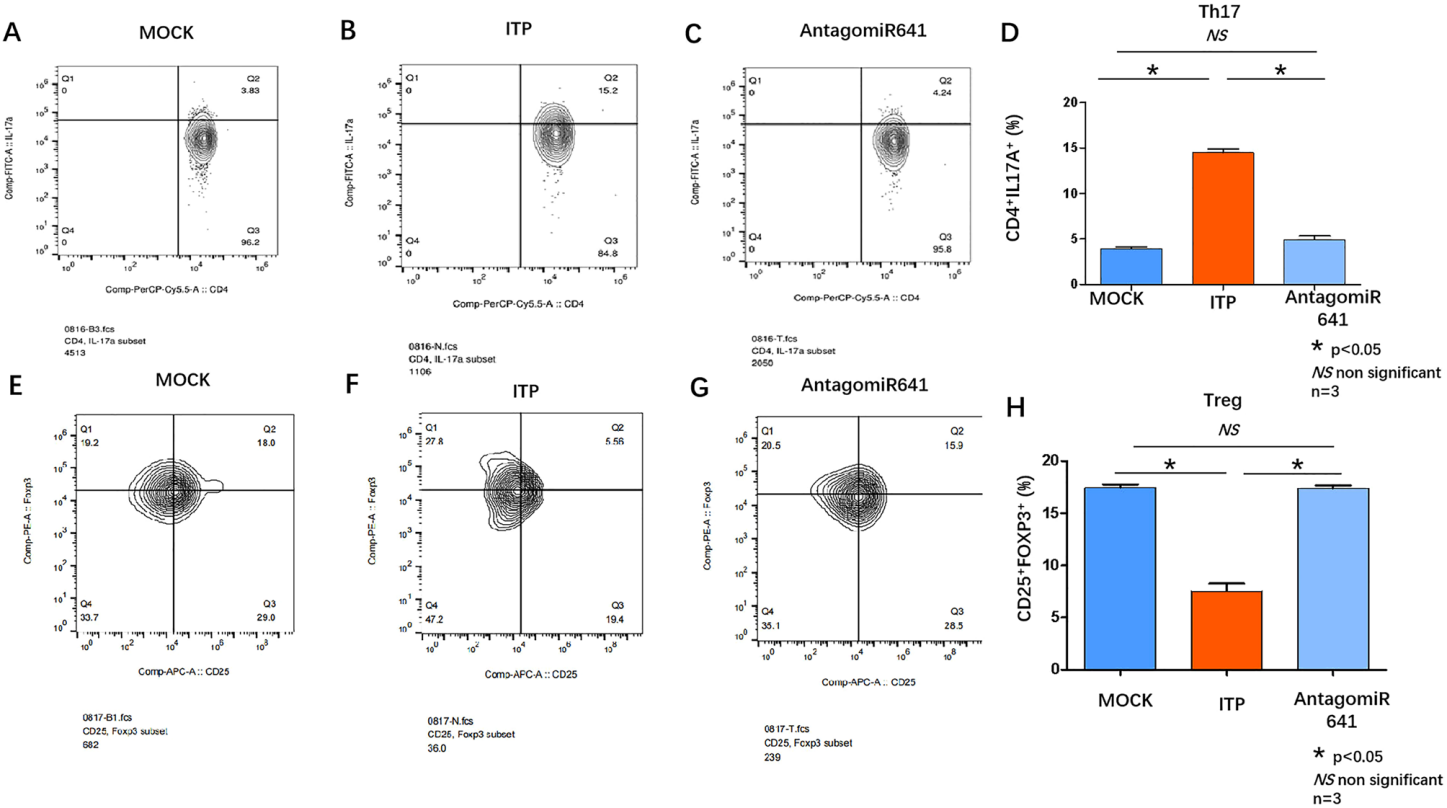
(**A**) FACS analyzed the Th17 cells surface markers CD4 and IL-17A expression in Peripheral blood from MOCK group. (**B**) FACS analyzed the Th17 cells surface markers CD4 and IL-17A expression in Peripheral blood from ITP group. (**C**) FACS analyzed the Th17 cells surface markers CD4 and IL-17A expression in Peripheral blood from AntagomiR641 group. (**D**) Statistic analysis of FACS results: CD4^+^IL17A^+^ Th17 cells were up-regulated in peripheral blood from ITP group (red column) comparing that from MOCK group (dark blue column) . Administration of AntagomiR 641 could down-regulate the CD4^+^IL17A^+^ Th17 cells (light blue column). (**E**) FACS analyzed the Treg cells surface markers CD25 and Foxp3 expression in Peripheral blood from MOCK group. (**F**) FACS analyzed the Treg cells surface markers CD25 and Foxp3 expression in Peripheral blood from ITP group. (**G**) FACS analyzed the Treg cells surface markers CD25 and Foxp3 expression in Peripheral blood from AntagomiR 641 group. (**H**) Statistic analysis of FACS results: CD25^+^Foxp3^+^ Treg cells were down-regulated in peripheral blood from ITP group (red column) comparing that from MOCK group (dark blue column) . Administration of AntagomiR 641 could restore the CD25^+^Foxp3^+^ Treg cells (light blue column). All FACS analysis underwent biological replication (n = 3).

Flow cytometry revealed that the proportion of the Th17 subpopulation was upregulated in the ITP group (14.52 ± 0.36) compared with the MOCK group (3.92 ± 0.23), while it was downregulated in the AntagomiR-641 group (4.94 ± 0.42) (Fig. 6A,B,C,D). The proportion of Tregs was lower in the ITP group(7.52 ± 0.26) than in the MOCK group (17.43 ± 0.32), while it was greater in the antagomiR-641 group (17.40 ± 0.26) than in the ITP group (Fig. 6E,F,G,H).

**Figure 7:**
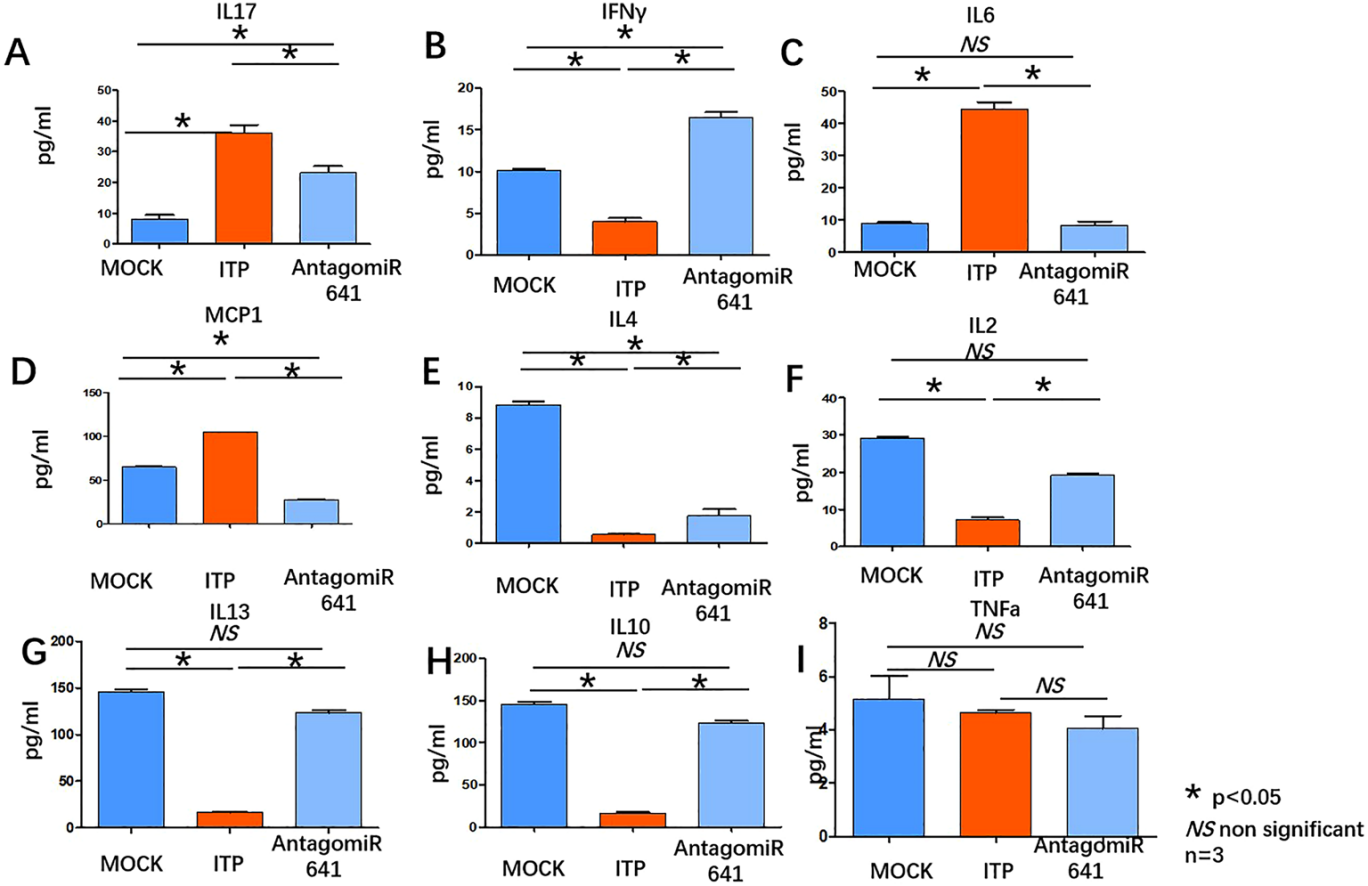
(**A**–**I**) Merck Millipore liquid phase microarray assays were applied for plasma cytokines. IL17, IFNγ, IL6, MCP1, IL4, IL2, IL13, IL10, TNFa were determined in MOCK group (dark blue column), ITP group (red column) and AntagomiR641 group (light blue column) respectively. Cytokine measurement underwent biological replication (n = 3) .

Cytokine assays revealed that IL17A promoted Th17 differentiation and was downregulated in the antagomiR-641 group compared with the ITP group but did not reach the level of the MOCK group (Fig. 7A). Compared with that in the ITP group, IFNγ downregulated Th17 and upregulated Treg cell numbers and was upregulated in the AntagomiR-641 group, and IFNγ expression was upregulated in the AntagomiR-641 group compared with the MOCK group (Fig. 7B). IL6, a regulator of the Treg/Th17 balance, inhibits TGFβ-induced Treg differentiation and induces Th17 differentiation. This cytokine was downregulated in the AntagomiR-641 group compared to the ITP group and was comparable to that in the MOCK group (Fig. 7C). Th17 cells promoted the expression of MCP1, which was significantly downregulated in the AntagomiR-641 group compared with the ITP group and significantly inhibited compared with the MOCK group (Fig. 7D). IL4 inhibited Th17 differentiation, a cytokine that was upregulated in the AntagomiR-641 group compared to the ITP group but did not reach the expression level of the MOCK group (Fig. 7E). IL-2 promoted Treg differentiation, and this cytokine was upregulated in the AntagomiR-641 group compared to the ITP group, and the levels were comparable to those in the MOCK group (Fig. 7F). IL13 is a Th2-type cytokine that promotes Treg differentiation; this cytokine was upregulated in the AntagomiR-641 group compared to the ITP group, and its levels were comparable to those in the MOCK group (Fig. 7G) . Tregs secrete IL-10, which inhibits T-cell activation. This cytokine was upregulated in the AntagomiR-641 group compared to the ITP group and was comparable to that in the MOCK group (Fig. 7H). TNFa reportedly increases the number of Tregs and upregulates the expression of Foxp3 in patients with IBD. The results of this study revealed no significant difference in the expression of this cytokine between the AntagomiR-641 group and the ITP group or between the AntagomiR-641 group and the MOCK group, but there was no significant difference (Fig. 7I).

**Figure 8:**
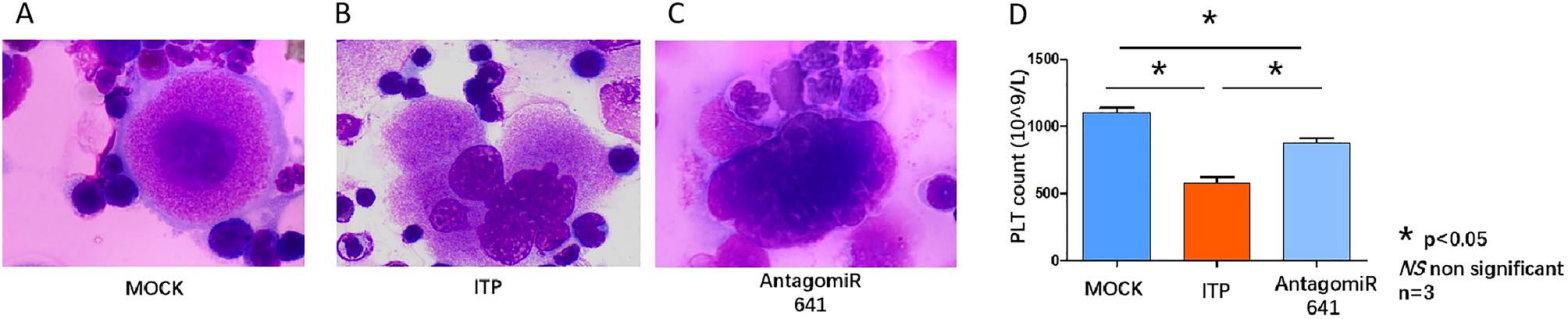
A, B, C. Giemsa staining were performed in bone marrow smear from MOCK group (**A**), ITP group (**B**) and AntagomiR641 group (**C**). Giemsa Staining underwent biological repilication (n = 3). (**D**) Platelet count were analyzed in MOCK group (dark blue column), ITP group (red column) and AntagomiR641 group (light blue column).Platelet count analysis underwent biological replication (n = 3).

According to Deka et al., megakaryocytes in ITP patients have a greater nuclear/cytoplasmic ratio, lower nuclear roundness factor and lower nuclear contour ratio. Cellular circularity and compactness were significantly different between ITP patients and non-ITP patients, suggesting that there were fewer megakaryocytes in ITP patients than in non-ITP patients^30^. In our study, bone marrow smears following Giemsa staining were used to observe the morphology of megakaryocytes in each group. Compared with those in the MOCK group, the megakaryocytes in the ITP group had a greater nuclear/cytoplasmic ratio, were less circular and increased in cell size; however, compared with those in the ITP group, the megakaryocytes in the AntagomiR-641 group had a decrease in cell size and more circular morphology but still had a greater nuclear/cytoplasmic ratio (Fig. 8A,B,C). The platelet counts suggested that the amount of platelets in the peripheral blood of the ITP group was greatly decreased compared with that in the peripheral blood of the MOCK group, while it was increased in the AntagomiR 641 group (Fig. 8D).

## Discussion

In the present study, we obtained the following findings.

1. miR-641 was upregulated in ITP patients. The interaction of miR-641 with SATB1 and STIM1 was observed by dual-luciferase reporter gene reporter assays. The overexpression of miR-641 in 293 T cells reduced SATB1 and STIM1 levels.
2. The overexpression of miR-641 in human primary T cells in vitro resulted in the downregulation of SATB1 and STIM1 expression and could cause the upregulation of Th17 cells and the downregulation of Treg cells.
3. Using a miR-641 sponge to block miR-641 expression in ITP patients’ primary T cells resulted in the upregulation of SATB1 and STIM1 expression, the downregulation of Th17 cells, and the upregulation of Treg cells.
4. In a murine ITP model, tail vein injection of antagomiR-641 downregulated peripheral miR-641 expression and upregulated SATB1 and STIM1 expression, thus restoring the Th17/Treg balance while also correspondingly regulating changes in peripheral plasma cytokine expression and increasing the PLT.

### Progression of ITP treatment

ITP is a tissue-specific autoimmune disease. Current knowledge of this disease suggests that its development is closely related to the dysfunction of immune cells such as T cells, B cells, dendritic cells and macrophages. First-line treatments for ITP include steroids, intravenous immunoglobulin (IVIg) and/or Rh immune globulin (anti-D). Second- and third-line treatments include roxan, fostamatinib, thrombopoietin receptor agonists (TPO-RAs), and/or splenectomy. Treatment failure in ITP patients is usually associated with severe immune system dysfunction^31,32^.

### The role of Treg/Th17 cells in the pathogenesis of ITP

T-cell immune abnormalities play a key role in the development of ITP. Th1, Th2, Th17, Th22, Tfh and plasma IL-22, IL-17A, and IFN-gamma expression was upregulated in the plasma of ITP patients. CD4^+^CD25^+^ Treg cells play an important role in maintaining peripheral immune tolerance and are significantly reduced in both the peripheral blood and bone marrow of ITP patients. Accumulating evidence emphasizes that targeting T cells might be a potential strategy for ITP treatment. Early in 2014, Ji and colleagues reported that haloguginone significantly recovered peripheral platelet counts in ITP mice by promoting Th1 cell differentiation and attenuating Th2 differentiation^4^. Many research groups have reported that restoring the Th17/Treg imbalance could achieve therapeutic benefits for ITP. Guo and colleagues reported that resveratrol couldplay a therapeutic role in ITP by restoring the Th17/Treg imbalance^9^. Indirubin regulates CD4+ T-cell homeostasis in ITP via the PD1/PTEN/AKT signaling pathway^12^. Dexamethasone modulates the polarization of Th17 cells to CD4+ T cells in patients with primary ITP^14^. Halofuginone has also been reported to exert a therapeutic effect by modulating T-cell function in a mouse model of ITP^33^.

In addition to traditional drug therapy, research into the role of noncoding RNAs in the pathogenesis of ITP has been intensively conducted in recent years. The long noncoding RNA MEG3 inhibits microRNA-125a-5p expression and induces an imbalance of Treg/Th17 cells in ITP^34^. Hua and colleagues reported that the expression of microRNAs in CD4^+^ cells contributes to Th17/Treg imbalance in ITP. They found that miR99a expression was lower in ITP patients, while miR-183-5p and miR-183-5p were more highly expressed in ITP patients^11^. Li et al. reported that miR-106p-5p induces an imbalance of Treg/Th17 cells in ITP through the NR4A3/Foxp3 pathway^21^. Decreasing lncRNA PVT1 causes Treg/Th17 imbalance via NOTCH signaling in ITP^20^.

### Functions of miR-641

Previous studies have shown that miRNA641 is involved in regulating the progression of a variety of solid tumors by regulating the expression of its target genes. Intronic miRNA-641 controls its host gene pathway PI3K/AKT, and this relationship is dysfunctional in glioblastoma multiforme^35^. miR-641 Functions as a Tumor Suppressor by Targeting MDM2 in Human Lung Cancer^36^. The lncRNA TUSC8 inhibits the invasion and migration of cervical cancer cells via the miR-641/PTEN axis ^37^. However, the role of miR-641 in the regulation of immune system function has not been reported. Lamya Garabet et al. reported the changes in the expression levels of circulating miRNAs in ITP patients before and after TPO-RA treatment^38^. The authors screened out six miRNAs that showed significant changes, but our reported miR641 was not included. This could be due to different observation subjects: this study observed the differences in miRNAs between ITP patients and normal healthy controls, while Dr. Lamya Garabet's team observed the differences in miRNAs in ITP patients before and after TPO-RA treatment. On the other hand, we collected T cells from ITP patients as the observation subjects, while Dr. Lamya Garabet's team observed circulating miRNAs without collecting T cells. In the present study, we elucidated for the first time the important regulatory role of miRNA641 in ITP progression through a series of experimental observations. miR-641, discovered to have significant expression differences between T cells of ITP patients and healthy individuals, holds great potential as a biomarker for ITP. However, it is regrettable that due to the limited sample size of our current study, we are unable to draw a definitive conclusion. We hope to continue collecting specimens from ITP patients in further research and monitor their miR-641 expression levels, in order to make a more solid conclusion.

### STIM1 and T-cell development

Ca2 + channels are essential for T-cell development and activation, and STIM1 has been shown to be an important regulator of intracellular Ca2 + in T cells.However, the role of STIM1 in T-cell development is controversial. Samakai et al. reported that severe developmental defects were observed in thymocytes lacking PLC-gamma, which requires STIM1 to maintain Ca2+ signals^39^. Oh Hara et al. reported that STIM1 was dispensable for early thymic development based on relatively small differences in the numbers of DN, DP and SP cells^40,41^.

### STIM1 and T-cell activation

STIM1 is an important regulator in the early stages of T-cell activation. Calcineurin-dependent NFAT activation is dependent on the STIM1-mediated increase in cytosolic Ca2+. It is critical for regulating T-cell proliferation and the expression of activation-associated genes and effector functions such as the production of cytokines and chemokines^42^. STIM1 not only is required for Ca2+ entry but also modulates Ca2+ clearance in activated T cells^40^.

### Role of SATB1 in the regulation of T-cell function

SATB1 mediates the Wnt signaling pathway by recruiting β-catenin to its target genes and regulates Th2 differentiation through the upregulation of GATA-3. SATB1-deficient mice have disproportionately small thymuses, spleens and lymph nodes, suggesting irreversible damage to thymocyte development and function, and these mice usually die at approximately three weeks of age^43^. SATB1 deficiency leads to the inactivation of Treg cell-specific superenhancers (Treg-SEs), resulting in Treg dysfunction. SATB1-dependent activation of Treg-SE is essential for the lineage specificity of Treg cells in the thymus. Inactivation of this pathway leads to the development of autoimmune-related diseases^44^. Schumann et al. applied a Cas9 ribonucleic acid protein (RNP) screen to identify key proteins that regulate TFS in Treg cells. In addition to Foxp3, PRDM1, Foxo1 and IRF4, SATB1 is important for Treg-mediated immunosuppression^45^. SATB1-deficient thymocytes could lead to inappropriate T lineages after both MHC class I- and II-mediated selection and fail to generate NKT and Treg subsets. SATB1 shapes the primary T-cell pool by directing lineage-specific transcriptional programs in the thymus^46^. This evidence emphasized the important regulatory role of SATB1 in T-cell development.

## Conclusion

1. For the first time, miR-641 expression in T cells from ITP patients was described, and its interactions with the target genes SATB1 and STIM1 were screened and confirmed.
2. miR-641-SATB11 and STIM1 were found to play important roles in Th17/Treg regulation in the peripheral blood T cells of normal individuals and ITP patients.
3. Therefore, targeting the miR-641-SATB1/STIM1 axis might be a potential strategy for ITP therapy.

### Supplementary Information

Supplementary Information.

## Data Availability

All data generated or analysed during this study are included in this article.

## References

[1] Malik A, Sayed AA, Han P (2023). The role of CD8+ T-cell clones in immune thrombocytopenia. Blood.

[2] Audia S, Mahevas M, Samson M, Godeau B, Bonnotte B (2017). Pathogenesis of immune thrombocytopenia. Autoimmun. Rev..

[3] Yazdanbakhsh K, Provan D, Semple JW (2023). The role of T cells and myeloid-derived suppressor cells in refractory immune thrombocytopenia. Br. J. Haematol..

[4] Ji X, Zhang L, Peng J, Hou M (2014). T cell immune abnormalities in immune thrombocytopenia. J. Hematol. Oncol..

[5] Sun M, Wang X, Zhang N (2023). Imbalance of follicular regulatory T (Tfr) cells/follicular helper T (Tfh) cells in adult patients with primary immune thrombocytopenia. Exp. Biol. Med. (Maywood).

[6] Lin X, Xu A, Zhou L (2021). Imbalance of T lymphocyte subsets in adult immune thrombocytopenia. Int. J. Gen. Med..

[7] Kostic M, Zivkovic N, Cvetanovic A, Marjanovic G (2020). CD4(+) T cell phenotypes in the pathogenesis of immune thrombocytopenia. Cell Immunol..

[8] Chen Y, Zhou Y, Chen P, Zhang P, Jia M, Tang Y (2019). Excessive expressions of T cell activation markers in pediatric immune thrombocytopenia. Thromb. Res..

[9] Guo NH, Fu X, Zi FM, Song Y, Wang S, Cheng J (2019). The potential therapeutic benefit of resveratrol on Th17/Treg imbalance in immune thrombocytopenic purpura. Int. Immunopharmacol..

[10] Gu H, Chen Z, Shi X (2021). Increased proportion of Th17/Treg cells at the new diagnosed stage of chronic immune thrombocytopenia in pediatrics: the pilot study from a multi-center. Eur. J. Pediatr..

[11] Hua M, Li J, Wang C (2019). Aberrant expression of microRNA in CD4(+) cells contributes to Th17/Treg imbalance in primary immune thrombocytopenia. Thromb. Res..

[12] Zhao Y, Han P, Liu L (2019). Indirubin modulates CD4(+) T-cell homeostasis via PD1/PTEN/AKT signalling pathway in immune thrombocytopenia. J. Cell Mol. Med..

[13] Zhang A, Ning B, Sun N, Wei J, Ju X (2015). Indirubin increases CD4+CD25+Foxp3+ regulatory T cells to prevent immune thrombocytopenia in mice. PLoS One.

[14] Li J, Hua M, Hu X (2020). Dexamethasone suppresses the Th17/1 cell polarization in the CD4(+) T cells from patients with primary immune thrombocytopenia. Thromb Res.

[15] Wei Y, Ji XB, Wang YW (2016). High-dose dexamethasone vs prednisone for treatment of adult immune thrombocytopenia: A prospective multicenter randomized trial. Blood.

[16] Tan JH, Ahmad Azahari AHS, Ali A, Ismail NAS (2023). Scoping review on epigenetic mechanisms in primary immune thrombocytopenia. Genes (Basel).

[17] Zhao Y, Cui S, Wang Y, Xu R (2022). The extensive regulation of MicroRNA in immune thrombocytopenia. Clin. Appl. Thromb Hemost.

[18] Li W, Lv Y, Sun Y (2023). Roles of non-coding RNA in megakaryocytopoiesis and thrombopoiesis: new target therapies in ITP. Platelets.

[19] Mehana NA, Ghaiad HR, Hassan M, Elsabagh YA, Labib S, Abd-Elmawla MA (2022). LncRNA MEG3 regulates the interplay between Th17 and Treg cells in Behcet's disease and systemic lupus erythematosus. Life Sci..

[20] Yu L, Zhang L, Jiang Z, Yu B (2021). Decreasing lncRNA PVT1 causes Treg/Th17 imbalance via NOTCH signaling in immune thrombocytopenia. Hematology.

[21] Li JQ, Tian JM, Fan XR (2020). miR-106b-5p induces immune imbalance of Treg/Th17 in immune thrombocytopenic purpura through NR4A3/Foxp3 pathway. Cell Cycle.

[22] Vrbensky JR, Nazy I, Clare R, Larche M, Arnold DM (2022). T cell-mediated autoimmunity in immune thrombocytopenia. Eur. J. Haematol..

[23] George JN, Woolf SH, Raskob GE (1996). Idiopathic thrombocytopenic purpura: a practice guideline developed by explicit methods for the American Society of Hematology. Blood.

[24] Neunert C, Lim W, Crowther M (2011). The American Society of Hematology 2011 evidence-based practice guideline for immune thrombocytopenia. Blood.

[25] Neunert CE, Cooper N (2018). Evidence-based management of immune thrombocytopenia: ASH guideline update. Hematol. Am. Soc. Hematol. Educ. Program.

[26] Neunert C, Terrell DR, Arnold DM (2019). American Society of Hematology 2019 guidelines for immune thrombocytopenia. Blood Adv..

[27] Teeling JL, Jansen-Hendriks T, Kuijpers TW (2001). Therapeutic efficacy of intravenous immunoglobulin preparations depends on the immunoglobulin G dimers: studies in experimental immune thrombocytopenia. Blood.

[28] Li X, Wang SW, Feng Q (2019). Novel murine model of immune thrombocytopaenia through immunized CD41 knockout mice. Thromb. Haemost..

[29] Krutzfeldt J, Rajewsky N, Braich R (2005). Silencing of microRNAs in vivo with 'antagomirs'. Nature.

[30] Deka L, Gupta S, Gupta R, Pant L, Kaur CJ, Singh S (2013). Morphometric evaluation of megakaryocytes in bone marrow aspirates of immune-mediated thrombocytopenic purpura. Platelets.

[31] Semple JW, Rebetz J, Maouia A, Kapur R (2020). An update on the pathophysiology of immune thrombocytopenia. Curr Opin Hematol.

[32] Despotovic JM, Grimes AB (2018). Pediatric ITP: Is it different from adult ITP?. Hematol. Am. Soc. Hematol. Educ. Program.

[33] Jin C, Jia Y, Jin C (2014). Therapeutic effect of Halofuginone on ITP mice by regulating the differentiation of Th cell subsets. Int. Immunopharmacol..

[34] Li JQ, Hu SY, Wang ZY (2016). Long non-coding RNA MEG3 inhibits microRNA-125a-5p expression and induces immune imbalance of Treg/Th17 in immune thrombocytopenic purpura. Biomed. Pharmacother..

[35] Hinske LC, Heyn J, Hubner M, Rink J, Hirschberger S, Kreth S (2017). Intronic miRNA-641 controls its host Gene's pathway PI3K/AKT and this relationship is dysfunctional in glioblastoma multiforme. Biochem. Biophys. Res. Commun..

[36] Kong Q, Shu N, Li J, Xu N (2018). miR-641 functions as a tumor suppressor by targeting MDM2 in human lung cancer. Oncol. Res..

[37] Zhu Y, Liu B, Zhang P, Zhang J, Wang L (2019). LncRNA TUSC8 inhibits the invasion and migration of cervical cancer cells via miR-641/PTEN axis. Cell Biol. Int..

[38] Garabet L, Ghanima W, Rangberg A (2020). Circulating microRNAs in patients with immune thrombocytopenia before and after treatment with thrombopoietin-receptor agonists. Platelets.

[39] Samakai E, Hooper R, Soboloff J (2013). The critical role of STIM1-dependent Ca2+ signalling during T-cell development and activation. Int J Biochem Cell Biol.

[40] Samakai E, Hooper R, Martin KA (2016). Novel STIM1-dependent control of Ca2+ clearance regulates NFAT activity during T-cell activation. FASEB J..

[41] Oh-Hora M, Yamashita M, Hogan PG (2008). Dual functions for the endoplasmic reticulum calcium sensors STIM1 and STIM2 in T cell activation and tolerance. Nat. Immunol..

[42] Feske S (2007). Calcium signalling in lymphocyte activation and disease. Nat. Rev. Immunol..

[43] Burute M, Gottimukkala K, Galande S (2012). Chromatin organizer SATB1 is an important determinant of T-cell differentiation. Immunol. Cell Biol..

[44] Kitagawa Y, Ohkura N, Kidani Y (2017). Guidance of regulatory T cell development by Satb1-dependent super-enhancer establishment. Nat. Immunol..

[45] Schumann K, Raju SS, Lauber M (2020). Functional CRISPR dissection of gene networks controlling human regulatory T cell identity. Nat. Immunol..

[46] Kakugawa K, Kojo S, Tanaka H (2017). Essential roles of SATB1 in specifying T lymphocyte subsets. Cell Rep..

