# Supplementary material for: The miR-641-STIM1 and SATB1 axes play important roles in the regulation of the Th17/Treg balance in ITP
Source: Sci Rep. 2024 May 16;14:11243. doi: 10.1038/s41598-024-61660-9 (PMC11098809; doi:10.1038/s41598-024-61660-9)
Supplement: Supplementary file 1 — Supplementary Information. [file 41598_2024_61660_MOESM1_ESM.docx]

**Table S1 Primers used in qRT-PCR**

| **Gene** | **Forwoard** | **Reverse** |
| --- | --- | --- |
| SATB1 | TCAGACATCTCCTGTAGGGC | GTGGACCCTTCGGATCACTC |
| STIM1 | TCCCCAGCTTCTGCTGCTC | CCGCAGATTACTACACCCCTG |
| TGFBR1 | CCTCGAGATAGGCCGTTTGT | AAGGGCCAGTAGTTGGAAGTT |
| DDX5 | CCGACCAAAACCCGTCAAA | GAGGAAGGACACCGATGACAC |
| miR641 | miDETECT A TrackTM hsa-miR-641 （rmiRA1001292，RiboBio Inc.） |  |

**Table S2 has-mir-641 over expression plasmid information**

**Plasmid**

pHBLV-U6-MCS-CMV-ZsGreen-PGK-PURO

**Target Sequence**

**>hsa-mir-641 MI0003656**

UGGGUGAAAGGAAGGAAAGACAUAGGAUAGAGUCACCUCUGUCCUCUGUCCUCUACCUAUAGAGG

UGACUGUCCUAUGUCUUUCCUUCCUCUUACCCCU

**Replace U with T**

TGGGTGAAAGGAAGGAAAGACATAGGATAGAGTCACCTCTGTCCTCTGTCCTCTACCTATAGAGGTG

ACTGTCCTATGTCTTTCCTTCCTCTTACCCCT

**Genome**

tgagcatatactctgcccagccctgtgctttgggctttatatggtatttgtgtccttttttgcagagtccgaaattgaagttcagaggaagaattgccttccaccgcatcttatca

gaccctatgggtgccagggtaggatttgaacccaggtcactgagcccctagtgccatgcattccttgctcaccaggttccaggctgggtgaaaggaaggaaagacata

ggatagagtcacctctgtcctctgtcctctacctatagaggtgactgtcctatgtctttccttcctcttacccctgagcctcaggctgccctgagcatctctgcctacagccag

tccagcatttcttggtccctcctcagagtggaggcaaggcatcctttaaccctgggcaggtgtgtgctgggagaagaagacagcaagagagccatgcagggggtgac

Acagctaccagggacctgcgttagctagcagcaggacggtgaagtcggttctag

**Primer**

| **Gene** | **Forwoard** | **Reverse** |
| --- | --- | --- |
| LV-hsa-mir-641-M | cctgaatctaggtcgacaatctgagcatatactctgcccagc | atccagaggttgattgttccagacgcgtctagaaccgacttcaccgtcc |

**Table S3 h-hsa-miR-641sponge plasmid information**

**Plasmid**

pHBLV-U6-MCS-CMV-ZsGreen-PGK-PURO

**Target Sequence**

>hsa-miR-641 MIMAT0003311

AAAGACAUAGGAUAGAGUCACCUC

**Replace U with T**

AAAGACATAGGATAGAGTCACCTC

**Reverse Complement**

GAGGTGACTCTATCCTATGTCTTT

**Primer**

| **Gene** |  |
| --- | --- |
| Lv-hsa-miR-641 sponge-Bam-F | acaggatccGAGGTGACTCTATCCTATGTCTTTtatacGAGGTGACTCTATCCTATGTCTTTacatcGAGGTGACTCTA |
| Lv-hsa-miR-641 sponge  Eco-R | acagaattcaaaaaaAAAGACATAGGATAGAGTCACCTCtgaagaAAAGACATAGGATAGAGTCACCTCgatgtAAAGA |

**Table S4 pmiR-RB-ReportTM 3'UTR reporter gene vector construction (STIM1)**

**Target sequence name**

h-STIM1(NM_001382578.1 3UTR:333-985)-WT

**Clone length**

653bp (NM_001382578.1 3UTR:333-985)

**5’ Restriction Enzyme cutting site** XhoI

**3’ Restriction Enzyme cutting site** NotI

**Vecotr name:** pmiR-RB-ReportTM

**Reportor gene:** Renilla Luciferase

**Rreference gene:** Firefly Luciferase

**Antibiotics resistance:** Ampicillin

**Clone fragment sequencing results**

GAGCGGCATGGATAGACACCCTGCTGCTTGCGCCAGCGCCAGGATCAACGTCTAATTCTAGGCGATCGCTCGAGAA

ACACACGCATTCCCCACCTGGCTGGCAAGAAGGCTGTGGCTGAGGAGGATAATGGCTCTATTGGCGAGGAAACAGA

CTCCAGCCCAGGCCGGAAGAAGTTTCCCCTCAAAATCTTTAAGAAGCCTCTTAAGAAGTAGGCAGGATGGGGTGGC

AGTAAAGGGACAGCTTGTCCTTCCCTGGGTGTTCTGTCTCTCCTTCCCTCCCTTCCTTCAAGATAACTGGCCCCAA

GAGTGGGGCATGGGAAGGGCTGGTCCAGGGGTCTGGGCACTGTACATACCTGCCCCCTCATCCTTGGGTCCTTCAT

TATTATTTATTAACTGACCACCATGGCCTGCCTGCCCTGCCTCCGTCCCAACCATGGGCTGCTGCTGTCACTCCCT

CTCCACTTCAGTGCATGTCTTAGTTGCTGTTCCCTCAGCTCCCAGCTCCACCTCTGGGGTTCAGCTTCTGTCTCTG

CTGTCCCAGTTTTGAGGTTTGGTTTCTTGTTTCTGTCTCTTGCTTTCAGGCTCCTCCCTCCCACCACTCCCCAACT

TCCCCTAGCAGTTGCAGGGAAGATAGGACGAGTAGCTTCTGACATGTGTGCCTCAGATCTGTTCCACCCCACTCAC

AGTGGTTCTGTTTGCTCCAGACTGGGGCTAGGGCCTAATCTTGCGGCCGCTGGCCGCAATAAAATATCTTTATTTT

The underlined sequence is the cloned target sequence, flanked by sequences from the vector.

Blue font represent SNP: 507bp G/A (Variation ID:rs1561876)

**Table S5 pmiR-RB-ReportTM 3'UTR reporter gene vector construction (SATB1)**

**Target sequence name**

h-SATB1(NM_001131010.4 3UTR:507-1024)-WT

**Clone length** 518bp (NM_001131010.4 3UTR:507-1024)

**5’ Restriction Enzyme cutting site** XhoI

**3’ Restriction Enzyme cutting site** NotI

**Vecotr name:** pmiR-RB-ReportTM

**Reportor gene:** Renilla Luciferase

**Rreference gene:** Firefly Luciferase

**Antibiotics resistance:** Ampicillin

**Clone fragment sequencing results**

h-SATB1(NM_001131010.4 3UTR:507-1024)-WT

CCGGCACCCTGCCCATGAGCTGCGCCCAGGAGAGCGGCATGGATAGACACCCTGCTGCTTGCGCCAGCGCCAGGAT

CAACGTCTAATTCTAGGCGATCGCTCGAGACATATGCCAACAAATGCCTTGTATTATATGGCACTGCCGTAATTCA

AATTTGTTTTTATTTTGGAAATAAAAGTTCACTGTACTTTTTTTTCATTCTCATTGTTACATGATTTTTTAAAAAA

AGGAAAAGAAAATGTGAAACACAATTTAGTCCTCATTATTTATTTGTAGATCCTGCAGCATCATGTTGTAATTAAT

TTTTTGGAAGTTTCCGTTAAATGTAATATTGCTTCTCTTGTTACCATACTGATTCTTTTCTATTTATAAATGTATT

TTGATGGGCAGTAAAACAAAGTGTCTTAAAAGTTTTAAATAGAGAAAATGTGCTTTACACAGTTGCCTATAAAAAG

TGCTCTATGTTATCCAAGCAATTCATACTATAAGCTTCACTCTTATTGTTGTATGCAATTTTTACTATCATGCAAA

TAAGCTTAGGTAAATAAAACTAATAGATCACCTTAGAAAATTATGCAATTAATGTGAAAATAATTGATGTTTGCAA

TGTGTCTTCCTTTGGCGGCCGCTGGCCGCAATAAAATATCTTTATTTTCATTAC

**
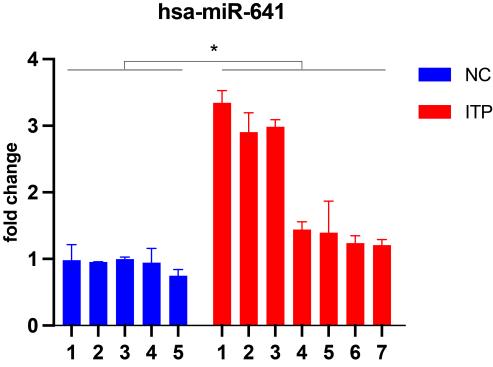
Figure S1**

****P*<0.05**

**Figure S2**

**
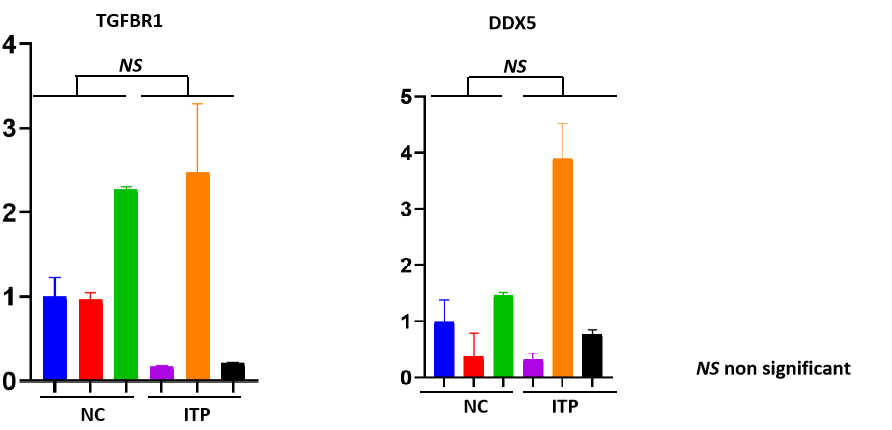
**

**
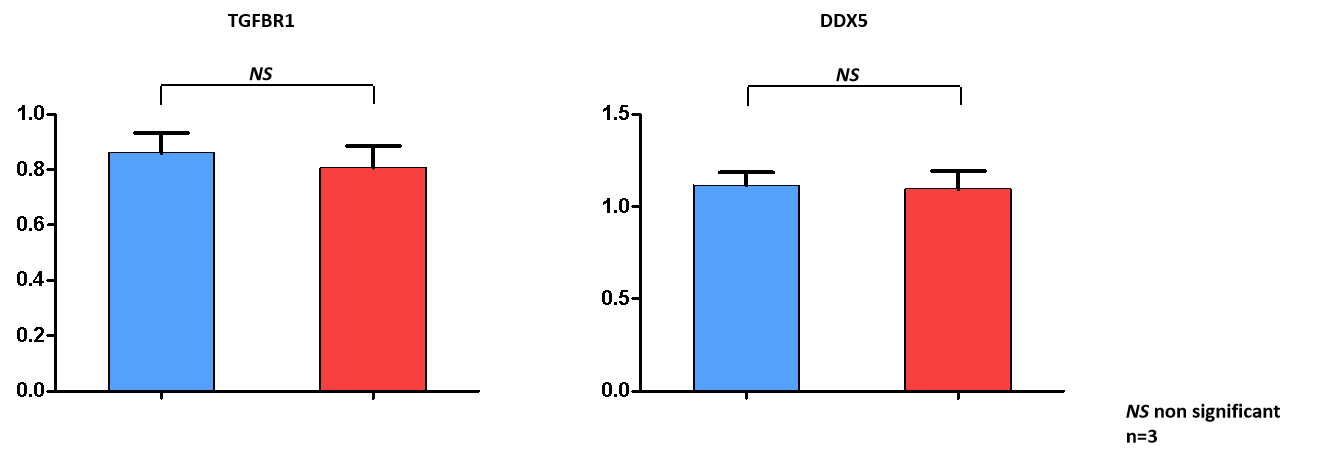
Figure S3**

**Figure Legends**

**Figure S1** qRT-PCR analyzed miR641 expression in normal control (NC, blue, n=5) and ITP patient (ITP, Red, n=7), * *p*<0.05. All groups underwent three technical replicates for qRT-PCR.

**Figure S2** qRT-PCR analyzed SATB1, DDX5 expression in normal control (NC, n=3) and ITP patients (ITP, n=3), *NS* non significant. All groups underwent three technical replicates for qRT-PCR. (Samples information for SATB1 & DDX5 were shown in Table 1)

**Figure S3** qRT-PCR analyzed SATB1, DDX5 expression in 293T cells after miR641 over-expression（lentivirus transfection), (Vecter, blue column; miR641 over -expression, Red column), *NS* non significant. All groups underwent three technical replicates for qRT-PCR.

**Western blotting raw data**

Vector

miR641OE

Vector

miR641OE


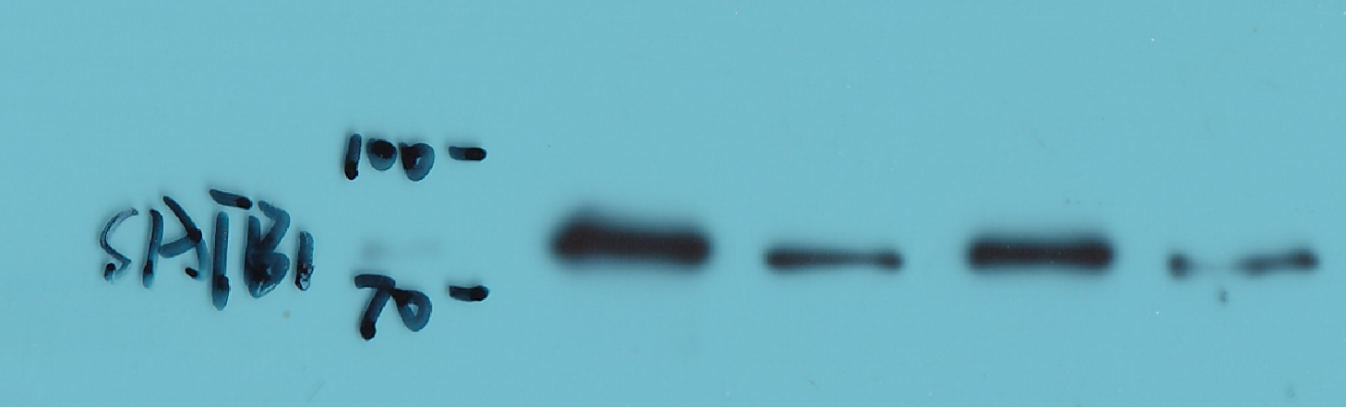


miR641

OE

Vector

Vector

miR641

OE


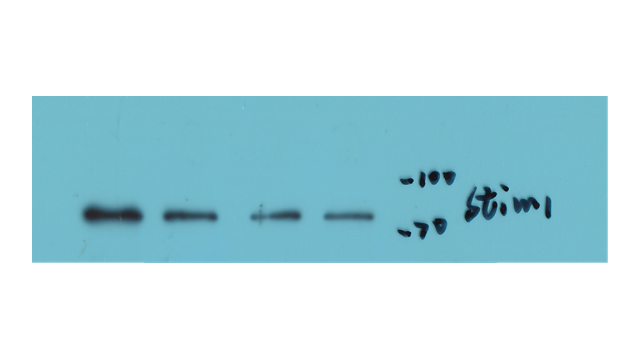


miR641

OE

Vector

miR641

OE

Vector


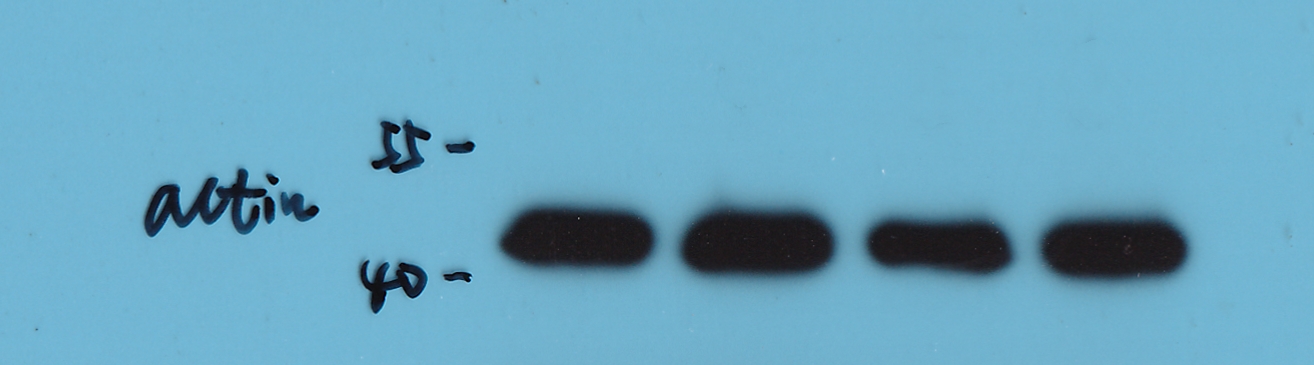


Due to the close proximity of the molecular weights of the two target proteins, SATB1 (86KD) and STIM1 (77KD), we cannot guarantee the incubation of both antibodies on the same PVDF membrane. Therefore, during the western blotting process, we conducted the experiment twice on separate PVDF membranes. We have provided the original, unmodified film data, and have marked the molecular weights and corresponding positions of the proteins using a marker pen.

During the Western blotting experiment, we used samples unrelated to this study on other lanes but shared the same antibodies. Therefore, we cropped the lanes of unrelated samples manually after exposure. To ensure the clarity and independence of the lanes in this experiment, we used blank buffers as separators between lanes. Consequently, there are indeed traces of cropping in the currently provided raw data. However, as the cropped film sections cannot be restored and were not properly preserved, and these samples are not directly related to this study, we sincerely hope that the raw data and explanations provided this time can be understood and accepted.
